# Unlimited Associative Learning and the origins of consciousness: a primer and some predictions

**DOI:** 10.1007/s10539-020-09772-0

**Published:** 2020-12-03

**Authors:** Jonathan Birch, Simona Ginsburg, Eva Jablonka

**Affiliations:** 1 Centre for Philosophy of Natural and Social Science, London School of Economics and Political Science, Houghton Street, London WC2A 2AE, UK; 2 Natural Science Department, The Open University of Israel, 1 University Road, 4353701 Raanana, Israel; 3 The Cohn Institute for the History and Philosophy of Science and Ideas, Tel Aviv University, 6934525 Ramat Aviv, Israel

**Keywords:** Consciousness, Learning, Unlimited associative learning, Evolution, Evolutionary transitions, Transition marker

## Abstract

Over the past two decades, Ginsburg and Jablonka have developed a novel approach to studying the evolutionary origins of consciousness: the Unlimited Associative Learning (UAL) framework. The central idea is that there is a distinctive type of learning that can serve as a transition marker for the evolutionary transition from non-conscious to conscious life. The goal of this paper is to stimulate discussion of the framework by providing a primer on its key claims (Part I) and a clear statement of its main empirical predictions (Part II).

## Introduction

A conscious system—an experiencing subject—has a subjective point of view on the world and on its own body. The system is capable of generating that elusive property that philosophers call phenomenal consciousness (Block 1995). It feels like something to be that system. Somewhere in nature there is a line between systems with no conscious experiences at all, like cyclones, stars and volcanoes, and those that do have at least some conscious experiences, like humans. Finding that line, and understanding how it was crossed, is a major challenge for science and philosophy (Godfrey-Smith 2019).

Over the past 2 decades, Ginsburg and Jablonka have developed a novel approach to studying the evolutionary origins of consciousness: the Unlimited Associative Learning (UAL) framework (Ginsburg and Jablonka 2007a, b, 2010a, b, 2015; Bronfman et al. 2016a, b). This work culminated in their recent book, *The Evolution of the Sensitive Soul: Learning and the Origins of Consciousness* (Ginsburg and Jablonka 2019). The approach, if it succeeds, promises to place origins of consciousness research on a firm theoretical and methodological footing, in a deliberate attempt to replicate the way origins of life research was placed on firmer foundations by the work of Gánti, Maynard Smith and Szathmáry. In a review of the book, Birch (2020) posed some problems for the UAL approach and highlighted the need for more explicit predictions. Here, our aim is to stimulate discussion of the UAL framework by providing a primer on its key claims (Part I) and a clear statement of its main empirical predictions (Part II).

## Part I: Primer

### Transition markers

At the heart of the UAL approach is the concept of an *evolutionary transition marker*. A transition marker is a property such that, when we find evidence of it, we have evidence that the major evolutionary transition in which we are interested has gone to completion.

The concept can be illustrated with the case of the origin of life. No one agrees precisely what life is. Nevertheless, there is a viable programme of origin of life research, built around a shared grasp of what it is the field aims to explain. There is *enough* consensus to make this possible, despite the lack of any consensus on a theory of the nature of life.

This is because there is consensus around a list of capacities that are jointly sufficient for life, capacities Gánti (2003) described as hallmarks of life. These capacities include maintenance of a boundary (individuation), metabolism, stability, information storage, regulation of the internal milieu, growth, reproduction, and death (eventual disintegration). The functional and structural coupling among the mechanisms and processes that implement these capacities constitute a living entity. There is some variation among the lists of hallmarks one finds in the literature, but a great deal of overlap.

When we have a list of capacities jointly sufficient for life, we can then ask: Is there some *single* positive marker that requires the existence of systems with *all* of these capacities? Gánti (2003) and Maynard Smith and Szathmáry (1995) proposed that there is: *unlimited heredity*. Unlimited heredity is the capacity to form lineages of open-ended length, varying in open-ended ways from the initial system. The DNA-based heredity system of all life on Earth is an example (and the only known biochemical example) of an unlimited heredity system. The point is not that DNA-based heredity operates entirely without constraint, but that the possibilities are sufficiently open-ended that there is no serious prospect of all of the possible morphological forms it can produce being explored by real lineages, even given a timescale on the order of the age of the universe.

A case can be made that there could be no unlimited heredity in the world without the existence of systems that possess all the hallmarks of life: individuation, metabolism, stability, information storage, regulation of the internal milieu, growth, reproduction, and death (Gánti 2003; Bronfman et al. 2016b). It is tempting here to object: What about viruses? But it is no problem for the claim that unlimited heredity is impossible without life if there are some non-living systems that possess unlimited heredity, provided their ability to realize this property is *dependent on a living host*, as is the case with viruses. A “virus-only” world, devoid of living hosts, is impossible. A lengthy, self-replicating RNA in an unstructured chemical “soup” will be ephemeral. An even stronger argument can be applied to a computer virus. The genetic algorithm that underlies its “replication” operates on a long sequence of digits, which constitute an unlimited heredity system. But this artificial heredity system is the product of a living system (a human designer), and a world in which artificial unlimited heredity systems evolve first, before biological unlimited heredity systems, is impossible. The key idea here is that we can distinguish between original (or primary) and derived (or secondary) ways of achieving unlimited heredity. The derived ways would not be possible unless the world had, at least at some point, contained the original way.

**Fig. 1:**
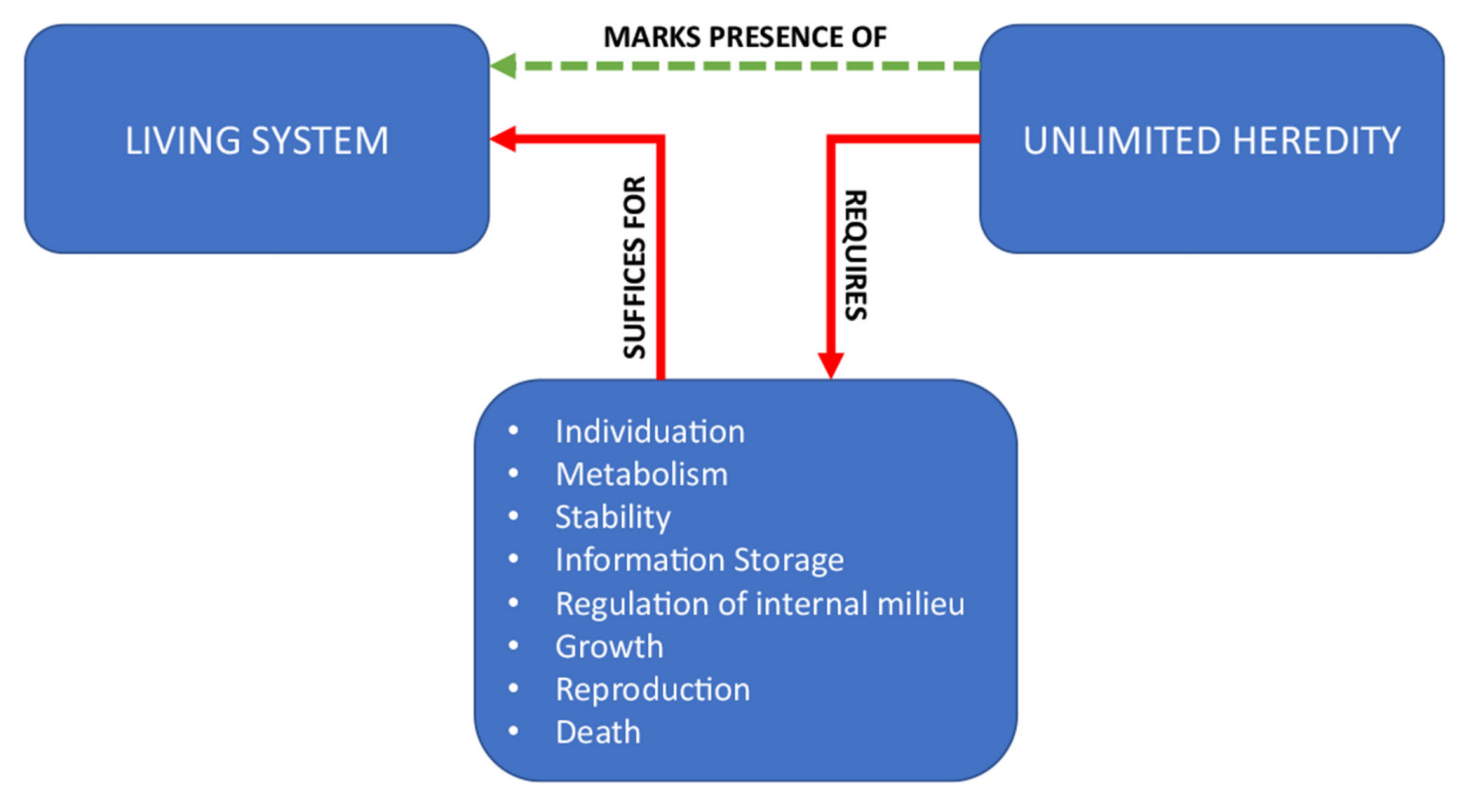
Unlimited heredity as a transition marker for the origin of life. Unlimited heredity requires (in its original, primary implementation) a set of capacities that suffice (given the actual laws of nature) for life

Granting that there could be no unlimited heredity without living systems, unlimited heredity provides a useful explanatory target for origin of life research. Researchers can ask: Can we date the origin of unlimited heredity to a particular geological period? Can we construct models of systems that are minimally sufficient to generate unlimited heredity?^1^ Can we model how those systems might have evolved? Agreeing on a transition marker allows theoretical and empirical research programmes to aim at a single, common goal, despite a potentially significant amount of underlying divergence in views about the fundamental nature of life (Fig. 1).

**Fig. 2:**
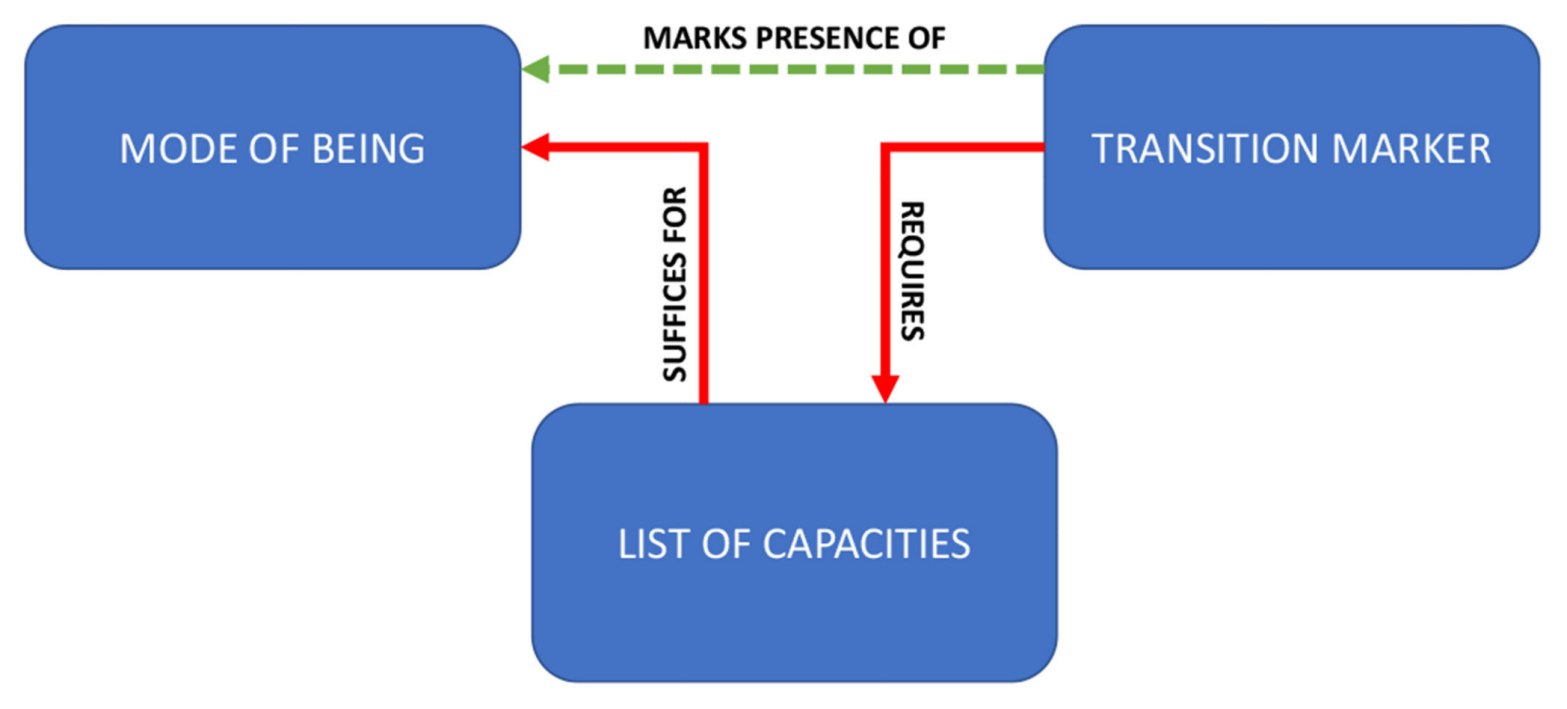
The general idea of a transition marker. A transition marker marks the presence of a mode of being (e.g. life, consciousness) by virtue of requiring (at least in its original, primary implementation) a set of capacities that suffice (given the actual laws of nature) for that mode of being

Life is not consciousness, but the case of life illustrates the general idea of a transition marker. In abstract terms, we can use the term “mode of being” to refer to the end-point of a major evolutionary transition. Life is a mode of being, and so is consciousness.^2^ To find a transition marker, we first to find a set of capacities that jointly *suffices,* given the actual laws of nature, for the biologically evolved mode of being we want to explain. We then identify a single property that requires, at least in its original, primary implementation, the existence of systems with all of those capacities. That property is our transition marker (Fig. 2).

### Sufficient conditions for consciousness

How can this strategy be applied to the case of consciousness, or subjective experience? Here too, we find a wide variety of theories about the nature of the phenomenon of interest, and a glaring absence of consensus. Our first task is to find hidden consensus behind the apparent disagreement by identifying a list of capacities that consciousness researchers would generally regard as *jointly sufficient* for a system being an experiencing subject—a subject with a point of view on the world and on its own body.

Like Gánti, we can construct a plausible list of hallmarks. First, there is *global accessibility and broadcast*: a minimal global workspace (in the sense of Dehaene 2014) where information from perception, memory and evaluative systems is integrated and broadcast back to these and other systems.^3^ Second, there is *binding/unification and differentiation:* objects (e.g. a blue box) are perceived, not just fragmented features (boxness and blueness). Third, there is *selective attention and exclusion:* there are mechanisms for making some stimuli more salient than others. Fourth, there is *intentionality*: the capacity to represent the world and one’s own body. Fifth, there is *integration of information over time,* not just at a single time. Sixth, there is an *evaluative system*. Seventh, there is *agency and embodiment*. Eighth, and finally, there is *registration of a self/other distinction.* ^4^ These hallmarks can be characterised in neural, cognitive, behaviour and phenomenological terms, as described in Table 1, although (of course) they will only have phenomenological manifestations in animals that are conscious. Further elaboration of each of these hallmarks can be found in Ginsburg and Jablonka (2019, Ch. 3, 5 and 8).

The longer and more demanding the list, the more plausible it is that it *suffices* for consciousness. No one is saying here that any of these properties is *necessary* for consciousness. That is not the point. Even a panpsychist could accept the claim that the above list *suffices* for a system being an experiencing subject, although they will feel it goes far beyond what is necessary. Moreover, the relevant notion of “sufficiency” in this context is *sufficiency in living organisms given the actual laws of nature*. So our criteria are not intended to be sufficient in the sense of *metaphysical* sufficiency: sufficiency in all possible worlds. The relevant notion is what philosophers call *nomological* sufficiency, and it is relative to a specific material substrate—living organisms. The metaphysical possibility (or otherwise) of “zombies” is therefore irrelevant to the sufficiency claim.

The next question is: Is there some *single* positive marker that requires the existence of systems with *all* of these capacities? Here there is a temptation to appeal to some uniquely human trait, such as sophisticated language, cumulative culture, symbolic art, or the ability to report one’s experiences verbally. These could technically serve as transition markers, but they would be unambitious and of little use. In effect, a transition marker gives a “no later than” timepoint for dating the evolutionary origin of consciousness. These markers would say: the transition was completed no later than the origin of behaviourally modern *Homo sapiens*. They would therefore tell us nothing we don’t already know, since no one seriously doubts that behaviourally modern *Homo sapiens* post-dates the origin of consciousness.

The real question is: Can we identify a more ambitious, more useful transition marker that allows us to push the end-point of the transition further back in time? In other words: Can we find a property that requires all the above hallmarks of consciousness, yet is possessed by a wide range of non-human animals? A property that is, so to speak, “as simple as possible, but not simpler”?^5^

### Unlimited Associative Learning (UAL)

The central postulate of the UAL framework is that we can, and that the transition marker is *Unlimited Associative Learning* (UAL). What is Unlimited Associative Learning? In broad terms, it is the within-lifetime analogue of unlimited heredity. A system with a capacity for unlimited heredity can give rise to lineages of open-ended future variation and open-ended evolution. A system with a capacity for UAL can, within its own lifetime, learn about the world and about itself in an open-ended way. As with unlimited heredity, the point is not that the process is entirely free of constraint, but that the possibilities are sufficiently open-ended that there is no serious prospect of all of the possible associative links it can produce being formed by a real organism with a realistic, finite lifespan.

We can be more precise about what UAL involves. It is associative learning with five crucial features that distinguish it from more limited forms:

1. *Compound stimuli* The conditioned stimulus can be a *compound* of discriminable perceptual features arranged in a pattern (e.g. a black-and-yellow buzzing object with a particular odour). These features may be in different sense modalities or in a single sense modality.
2. *Novel stimuli* The conditioned stimulus can be *novel* to the animal, in the sense that it is neither reflex eliciting nor pre-associated with an unconditioned stimulus or with past reinforcement. Moreover, the stimulus can be both novel and compound (e.g. a novel, complex pattern).
3. *Second-order conditioning* There is *second-order* as well as first-order conditioning. A conditioned stimulus can be associated with some other novel, compound conditioned stimulus or action, and so on, allowing the organism to build up long chains of associative links between stimuli and actions in an open-ended way.
4. *Trace conditioning* there can be a time gap (and no overlap) between the conditional and unconditional stimulus. There is an escape from immediacy, since the organisms can learn how stimuli that are no longer present relate to current stimuli.
5. *Flexible, easily rewritable associations with value* the positive or negative value of a stimulus or action can change quickly and flexibly in response to changes in the world. If a reinforcer is devalued (as in “outcome devaluation” paradigms; Holland and Rescorla 1975; Adams and Dickinson 1981), the animal will quickly adapt.^6^

Part of the UAL framework is that these enhanced forms of associative learning form a *natural cluster*. They have some overlapping features and are therefore not completely separate capacities which evolved completely independently from each other. Let us take the capacity for discrimination learning of novel, compound stimuli (features 1 and 2). In realistic ecological conditions, such learning involves pattern completion. Pattern completion is a form of associative learning: the ability to associate a partial pattern with the completed pattern. Once the completed pattern is associated with a positive or negative valence, learning to associate other things with the partial pattern can be considered second-order conditioning (feature 3). Moreover, the ability to discriminate between patterns requires that a previously perceived pattern is kept in working memory for a period of time while another pattern is scrutinized (feature 4). And in real environments the relationship between a completed pattern and reward can change quickly (e.g. a food source can suddenly acquire a nearby predator), so features 1–4 will be of little use unless the associations with value thus formed can be rewritten (feature 5). The various elements of UAL do not *logically* imply each other but they do depend on each other in realistic ecological settings.

**Fig. 3:**
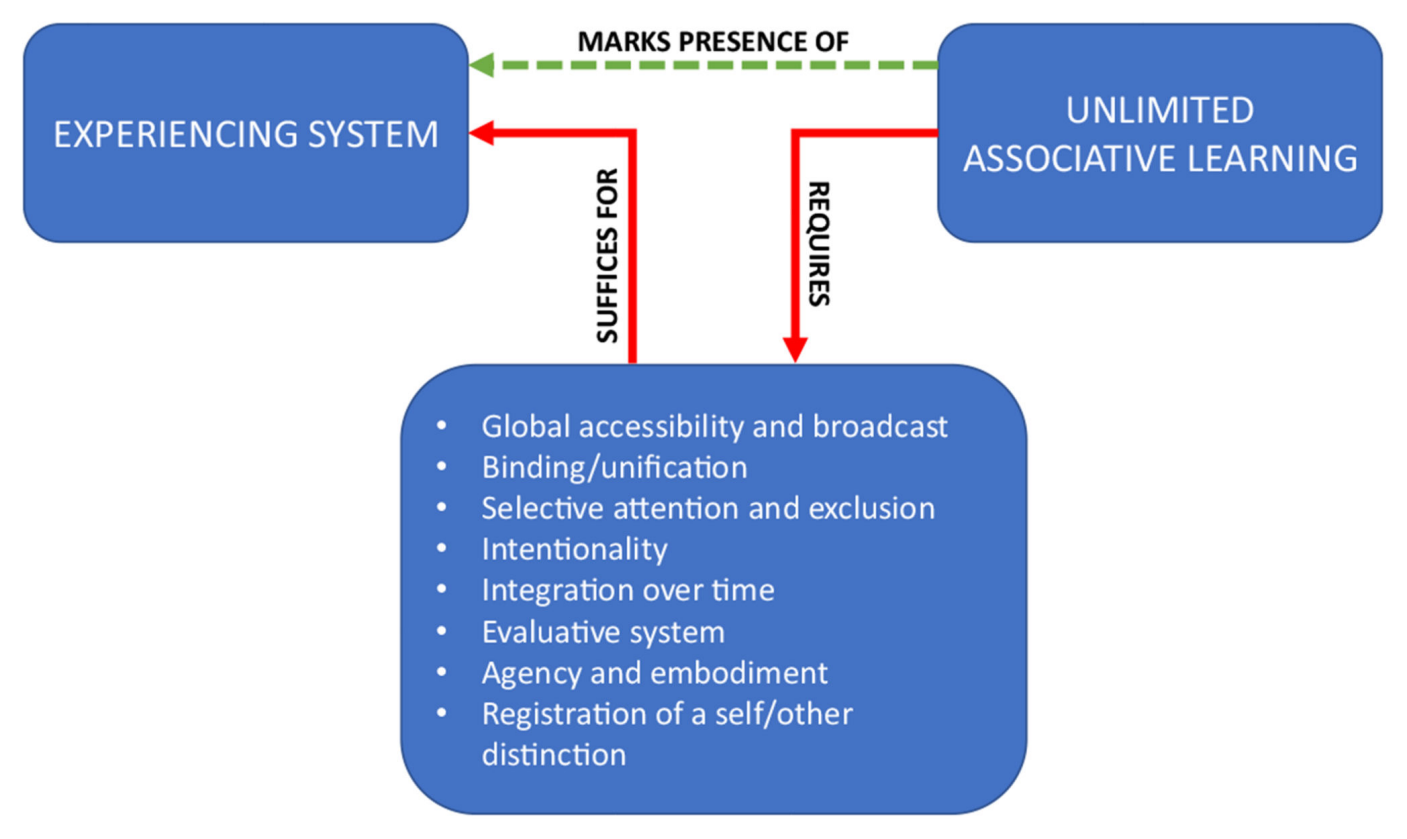
Unlimited Associative Learning (UAL) as a transition marker for the origin of consciousness

Why think that UAL, thus defined, is a good transition marker? Because it plausibly requires the existence of functionally coupled systems with all of the hallmarks of consciousness (Fig. 3). Some basic form of global accessibility and broadcast is plausibly needed to enable integration of information from multiple sense modalities. Some basic form of binding and differentiation is needed to construct compound stimuli. Some basic form of selective attention and exclusion is needed to pick these stimuli out from the background. Some basic form of intentionality is needed, since there must be some way in which the system represents stimuli when storing associative links. Some form of integration over time is needed to learn chains of associations between actions. Some basic form of evaluation system is needed to make classical conditioning and discrimination, based on reinforcement, possible at all. Some basic form of agency is needed for action selection, and thus making learning of associations between actions possible. Embodiment is needed to make agency possible. Finally, some basic form of self-other registration is needed so that the organism can distinguish between its own actions (and their “reafferent” effects on its sensory input) and stimuli generated by the external world, and thereby learn about the world and about the consequences of its own actions without conflating the two. In short, all the hallmarks of consciousness are needed (Bronfman et al. 2016a, b; Ginsburg and Jablonka 2019, Ch. 5). As in the case of life, the myriad of mechanisms underlying UAL in living organisms, constitute (are building blocks of and are nomologically sufficient for) biological consciousness. However, UAL is only a positive marker: it can tell us which animals are conscious, but it does not aspire to tell us which are not.

Just as one might be tempted to hold up viruses as a counterexample to unlimited heredity as a transition marker for life, one might be tempted to point to future or even current AI systems as counterexamples to UAL as a transition marker for consciousness. Isn’t it conceivable that AI could achieve genuinely open-ended associative learning with few, if any, of the hallmarks of consciousness in our list? But, as with viruses, this objection rests on a misunderstanding of what a transition marker is supposed to provide. It may be possible for conscious, embodied biological systems like *Homo sapiens* to design new, non-embodied, non-biological realizations of UAL which lack the hallmarks of consciousness. But these products of human intelligent design would not be the original, biologically evolved implementations of UAL, and they would owe their existence to these biologically evolved implementations. Their capacity to perform UAL would be derived from the capacity of an embodied, biological system to perform UAL. As a result, they are not a counterexample to the claim that there could be no UAL in a world in which experiencing subjects had never evolved, and they are not a counterexample to the claim that UAL can serve as a transition marker for the evolutionary origin of consciousness in living organisms.

UAL is well-equipped to play a role in origins of consciousness research that parallels that played by unlimited heredity in origins of life research. Researchers can ask: Which extant species are capable of UAL? Can we date the origin of UAL to a particular geological period? Can we construct models of systems that are minimally sufficient to generate UAL? Can we model how those systems might have evolved? Agreeing on a transition marker allows theoretical and empirical research programmes to aim at a single, common goal, despite a potentially significant amount of underlying divergence in views about the fundamental nature of consciousness.

### The distribution of UAL in the animal kingdom

To the best of our knowledge, which animals are capable of UAL? It is not easy to say at present, because animal cognition researchers have not been specifically looking for the whole UAL package. They have, however, looked for the individual elements of UAL in various species, and this allows for evidence-based conjectures about which taxa are most likely to possess the whole package.

Second-order conditioning, trace conditioning, and discriminative learning on novel, compound stimuli were found some time ago in rats, rabbits, pigeons, and goldfish.^7^ But, as far as we know, they were always studied separately: an ability to do second-order trace conditioning on compound stimuli was never directly tested. What we can say at the moment is that it seems very plausible that these animals possess the whole UAL package, given that they can demonstrate separate elements of the package on separate occasions.

Much the same can be said of honey bees (*Apis* genus) and fruit flies (*Drosophila* genus). Honey bees and fruit flies can learn associations between novel, compound visual stimuli (Couvillon and Bitterman 1988; Brembs and Heisenberg 2000; Schubert et al. 2002) and they can do second-order conditioning on olfactory stimuli (Hussaini et al. 2007; Tabone and de Belle 2011). There is also evidence of a simple form of trace conditioning on olfactory stimuli, though it is hard to be sure, with olfactory stimuli, that the conditioned stimulus has really gone by the time the unconditioned stimulus has arrived (Shuai et al. 2011; Szyszka et al. 2011; Dylla et al. 2013, 2017). So, we can credibly conjecture that they possess the whole UAL package, but we do not have hard evidence of this. As we argue in Part II, it is possible to test the relation between different aspects of UAL and consciousness. Yet, it remains conceivable that visual and olfactory learned discriminations rely on distinct mechanisms that both fall short of UAL.

Based on a review of the animal learning literature, Ginsburg and Jablonka (2019) conjecture that UAL is present in most vertebrates, some cephalopod molluscs (the coleoid cephalopods: octopods, squid and cuttlefish) and some arthropods (including honey bees and fruit flies). In these taxa it is also possible to identify the brain regions underlying processes of unification, dedicated memory systems for the storage of compound precepts, dedicated value systems, regions dedicated to motor programmes, and sensory-motor associative areas (Ginsburg and Jablonka 2019, Table 8.2).

On the basis of the literature, Ginsburg and Jablonka conjecture that UAL is absent in other invertebrate taxa, including gastropod molluscs (such as *Aplysia* sea slugs), annelids and nematodes. There is associative learning in these taxa, but it seems likely to be too limited to count as UAL. However, as they caution, the learning abilities of many species in species-rich taxa like molluscs and annelids have not been sufficiently studied, so the distribution of UAL may be broader than currently assumed.

### Attempting to trace the origins of UAL and some of its evolutionary effects

One of the functions of a transition marker is to help us date (in a “no later than” fashion) the transition in question. If UAL is our transition marker, what does it tell us about the timing of the transition from non-conscious to conscious beings? Unless UAL is much more widespread in the animal kingdom than suggested above, it hints at three separate origin events for conscious experience: one in the vertebrate lineage, one in the arthropods, and one in the coleoid cephalopod molluscs. If UAL is present in all or most extant vertebrates, this points to an origin event in the vertebrate case no later than the origin of the vertebrates, in the Cambrian period. A similar hypothesis has been reached, via a very different route, by Feinberg and Mallatt (2016).

A further claim defended by Ginsburg and Jablonka (2019), logically independent of the core components of the UAL framework, is that associative learning, both limited and unlimited did not just originate in the Cambrian explosion, but was one of the major *driving forces* behind that explosion. The Cambrian explosion saw a sudden and dramatic diversification of animal forms. Over evolutionary time, lineages were rapidly discovering new niches and evolving to occupy them. What explains this rapid pace of change? Ginsburg and Jablonka’s hypothesis is that associative learning, a form of developmental plasticity with clear potential for generating adaptive novelty, enabled organisms to develop novel behaviours within a single lifetime. They learned to exploit new environmental resources and to colonize new niches. Their abilities to adapt through learning allowed them to be more effective predators, more discriminating mates, and more evasive prey. Associative learning, and especially UAL, exerted great selection pressure on interacting species, which had to evolve to cope with the formidable UAL animals, or perish. This led to co-evolutionary arms races that drove the dramatic adaptive diversification of the Cambrian.

However, UAL, like every major innovation, led to new challenging problems and therefore was likely to have driven the evolution of mechanisms that ameliorate them. An urgent problem that UAL created was over-learning: since similar patterns may have different valences, pattern completion may sometimes lead to many false alarms. For example, a partial pattern of vibrations may sometimes be associated with a predator and sometimes with a nonthreatening passing animal, but since flight is less costly than injury, overreaction to the unthreatening cue is inevitable (the “smoke detector principle”, as coined by Nesse 2001). Over-reaction due to over-learning implies that animals are more often stressed. Since stress is physiologically costly leading to a greater propensity for disease (e.g., Liu et al. 2017; Everly and Lating 2019), it is expected that once associative learning and especially UAL evolved, ameliorating mechanisms such as the stress response, transgenerational inheritance of stress responses, and active forgetting, were more likely to evolve in UAL animals.

### Summary of the UAL framework

We will close this primer with a summary of the key claims of the UAL framework:

(0) In general, a *transition marker* is a property that requires a package of mechanisms and processes sufficient for a particular mode of being (e.g. life, consciousness), so that it provides a useful positive marker of when an evolutionary transition to that mode of being has gone to completion. Agreeing on a transition marker allows a research programme to unite around a shared set of questions (Can we date the marker to a particular geological period? Can we construct models of systems that are minimally sufficient to generate the marker? Can we model how those systems might have evolved?) despite substantial underlying disagreement about the fundamental nature of the mode of being in question.
(1) Unlimited Associative Learning (UAL) is the capacity for associative learning on novel, compound stimuli, with the potential for second-order conditioning and trace conditioning, allowing for the open-ended accumulation of long chains of associative links during an animal’s lifetime. It includes the ability to bridge temporal gaps: to learn about conditional stimuli that are no longer present. UAL is posited to be a natural cluster—a cluster of enhanced learning abilities that are closely linked.
(2) UAL is a transition marker for the transition to experiencing systems—systems with points of view on the world and on their own bodies, with states that feel like something to be in. This is because UAL, when implemented in a living organism, requires a package of underlying mechanisms and processes that are sufficient (given the actual laws of nature) for conscious experience.
(3) Given current evidence (which is admittedly limited) we can credibly conjecture that UAL (and thus conscious experience) is possessed by most vertebrates, some arthropods, and coleoid cephalopods. The framework does not rule out conscious experience in a wider range of invertebrates.
(4) This points towards an initial origin for UAL in the Cambrian period. This in turn leads to the conjecture that UAL, being a form of developmental plasticity particularly likely to generate adaptive novelty, played a role in driving the Cambrian explosion and drove the evolution of traits that partially compensated for its costs.

## Part II: Predictions

Part I highlighted five key claims of the UAL framework. One was that agreeing on a transition marker is an important step for origins of consciousness research, because it allows researchers to unite around a shared agenda despite substantial disagreement about the nature of consciousness (claim 0). This claim is not testable. However, the other four claims are all testable. In Part II, we ask: how can claims (1)–(4) be tested? What new data is needed to either confirm or refute the core commitments of the UAL framework?

### Testing Claim 1: UAL is a natural cluster

A core component of the UAL approach is that UAL is not just a list of five *independent* learning abilities that improve on more limited forms of associative learning. These abilities are taken to form a natural cluster—a cluster of correlated abilities which are enabled by the overlapping underlying mechanisms. This leads to three main predictions.

The first is that the five elements of UAL are ontogenetically correlated. Discrimination learning on novel, compound stimuli, second-order conditioning, trace conditioning, and flexible revaluing (and devaluing) of stimuli and actions are partially overlapping capacities, requiring many of the same mechanisms. This leads to the prediction that they will not develop completely independently of each other. The development of one of the five elements is expected to facilitate or enable the development of another element which may in turn affect the further development of the first element. The co-developmental relations may be different in vertebrates, arthropods and coleoid cephalopods.

The second is that the five elements of UAL are phylogenetically correlated. Due to the interdependencies between the elements explained in Part I, we expect that elements of UAL will be correlated in evolutionary terms. If a species is found to have evolved one of the elements of UAL (e.g. trace conditioning), this significantly increases the probability that it will also be found to have evolved the other elements (e.g., second-order conditioning). The prediction is that there will be substantial positive correlation between the elements, but the correlation need not be perfect. There could be some cases in which one or more of the elements of UAL evolved separately, without the rest of the package. The prediction is rather that the presence of one of the five elements raises the probability of finding the others. Substantial comparative data is needed to test this hypothesis: we first need to know the taxonomic distribution of the five elements of UAL in order to assess the correlation between these distributions.

The third prediction is that, in a UAL-possessing species, brain injuries that affect one element of the UAL cluster will affect some of the other elements. For example, hippocampal damage impairs trace conditioning in humans, rabbits and rats (Bangasser et al. 2006; Raybuck and Lattal 2014; McEchron et al. 1998; Moyer et al. 2015). The prediction is that, to the extent that trace conditioning is impaired, the other elements of UAL should also be impaired to some extent. Second-order conditioning and discrimination learning on novel, compound stimuli that require perceptual mapping and working memory are expected to be impaired. The elements of UAL should be, so to speak, medically correlated—damage that affects one part of the cluster should not leave the rest entirely unaffected, even though it may leave more limited forms of learning unaffected.

### Testing Claim 2: UAL is a transition marker for the transition to experiencing systems

The claim that UAL is a transition marker relies on the idea that a suite of mechanisms that (nomologically) suffices for subjective experience is also required (in biological systems) for UAL (Fig. 3). This leads to a basic prediction: if information about a stimulus does not reach the mechanisms in question, it will be neither subjectively experienced nor accessible to UAL. *Subjective experience of a stimulus and accessibility of that stimulus to UAL should come and go together*. This basic prediction is what we need to test, and the evidence must come from humans, because it is only in humans that we can independently verify, through verbal report, that a stimulus was consciously experienced. There are three main ways to test the basic prediction.

The first involves experimental protocols that manipulate whether or not a particular stimulus reaches subjective experience. Such protocols include backward masking, the attentional blink, and distracting tasks that lead to inattentional blindness (for examples and discussion see Dehaene 2014). The prediction is that, if the subject reports that they were never consciously aware of the stimulus—that they never experienced it—then *the forms of learning that constitute UAL will never be performed on that stimulus*, even though more limited forms of learning in relation to that stimulus may still be observed. For example, trace conditioning will be impossible, though delay conditioning may remain possible (Clark et al. 2002). Second-order conditioning of compound stimuli will be impossible, but first-order conditioning may still be observed. Discrimination learning involving simple stimuli may be observed, but discrimination learning when the stimulus is novel and compound will not be observed. Association of stimuli and actions with reward may still be observed, but rapid devaluation and revaluation—rapid rewriting of the links between stimulus, action and reward—will not be observed.

The second way involves subjects with blindsight, a condition that switches off conscious perception in a particular region of the visual field while leaving unconscious perception intact (Weiskrantz et al. 1974; Weiskrantz 2010). The prediction is that subjects who lack conscious perception in a given region of the visual field will be unable to perform UAL on any stimuli presented in that region, but more limited forms of associative learning may still be observed in relation to those stimuli. We should expect to find all the same dissociations between unlimited and limited forms of learning that we find in healthy subjects with masking and related protocols. Although blindsight is specifically a visual phenomenon, there is some evidence (albeit less compelling evidence) of related phenomena in other sense modalities (for hearing see Brogaard et al. 2017; for olfaction see Zucco et al. 2015).

The third path to relevant evidence involves the neural signatures of subjective experience. There is continuing debate about the neural signatures of experience in humans. Various potential neural signatures have been identified (reviewed in Dehaene 2014, Chapter 4). A specific type of neural oscillation (the gamma wave) has long been thought to be important, although not everyone agrees about this. A specific type of event-related potential (the P3 wave) also seems to matter. Conscious experience seems to result not from localised brain activity in a specific region, but rather from brain activity that implicates many different regions of the cortex, as well as the thalamus. Some take the prefrontal cortex at the front of the brain to play a special role (Dehaene 2014), whereas others emphasize a “posterior hot zone” at the back of the brain (Boly et al. 2017). Others highlight the significance of thalamocortical loops (e.g., Llinás 2003). Global ignition—the ultra-fast spread of activity across the entire cortex—has also been suggested as a potential signature (Noy et al. 2015).

To state our prediction, we don’t need to take sides on which of these are truly neural signatures of experience, or on the issue of whether the front or back of the neocortex is more important to subjective experience in humans. Our prediction is simply this: *the neural signatures that a stimulus is consciously represented* (*in a given taxon*) *will also be neural signatures of the accessibility of that stimulus to UAL* (*in a given taxon*). For example, if global ignition turns out to be an important neural signature of conscious representation of a stimulus in mammals, then global ignition will also be found when a stimulus is accessible to UAL. And this correlation is predicted to be robust across at least mammalian species, and perhaps more broadly: finding global ignition in a given species will be positively correlated with finding UAL in that species. To reiterate, however, the UAL framework does not make predictions at the level of specific neural signatures. What it predicts is that the neural signatures of experience, whatever they turn out to be, will also be correlated with UAL.

### Testing Claim 3: UAL (and thus conscious experience) is possessed by most vertebrates, some arthropods, and coleoid cephalopods

Although there is some evidence that UAL is present in most vertebrates, some arthropods and few molluscs, the precise distribution of UAL in these phyla is not known. There is huge variability in cognitive capacities among vertebrates, arthropods and molluscs, but only for a few species in each phylum do we have substantial data about their learning abilities (for a summary of the learning literature see Ginsburg and Jablonka 2019, chapter 8). For example, gastropod molluscs show rapid avoidance learning (Siegel and Jarvik 1974), and in the pond snail *Lymnaea stagnalis* second order conditioning of simple taste discrimination was observed (Sugai et al. 2006; for more tests see Benjamin and Kemenes 2010), but we are not aware of experiments testing for discrimination among compound stimuli by match to sample experiments. So, the claim that UAL is *absent* in these invertebrate taxa is very tentative and could easily be refuted by future experiments. It is only when animals can succeed in a variety of the tasks that test the different elements of UAL that we can positively ascertain that the animal has the capacity for UAL, and is, by implication, at least minimally conscious. Such tests may lead us to expand or narrow the range of species (and possible phyla) which display UAL.

### Testing (part of) Claim 4: UAL has trade-offs and drove the evolution of traits that partially compensated for its costs

In addition to the predictions testing the relation between UAL and consciousness, the UAL framework suggests some evolutionary-developmental predictions. Based on palaeontological evidence of well-preserved fossil brains of arthropods and vertebrates that exhibit the functional architecture of UAL, Ginsburg and Jablonka (2019) concluded that this form of learning evolved in these phyla during the mid-Cambrian from a simple form of elemental associative learning (called limited associative learning by Ginsburg and Jablonka 2019). The neural architecture of UAL suggests that its construction involved the evolution of dedicated memory structures for storing compound patterns of stimuli and actions, dedicated value systems, and a hierarchy of levels of sensory and motor integration units that enabled world and body mapping.

Associative learning was a leap in adaptability that can parsimoniously explain the major features of the Cambrian diversification (Ginsburg and Jablonka 2010b, 2019), but we think the assumption that associative learning *drove* the Cambrian explosion is not itself experimentally testable. What *is* testable is the hypothesis that UAL drove the evolution of mechanisms that partially compensated for the costs of over-learning engendered by UAL. These include mechanisms restricting the duration and effects of the stress response during ontogeny, mechanisms restricting the epigenetic inheritance of the effects of stress responses, and the evolution of active forgetting.

(I) *Co-evolution of learning and the stress response*: We predict that these mechanisms started co-evolving in animals showing limited associative learning and brain centralization. We therefore expect to find evidence for the co-evolution of the gene regulatory networks (GRNs) underlying associative learning and the stress response. We also expect that genes involved in sophisticated stress responses may have been lost in animals such as penis worms (which seem to have lost their brain during evolution, though they still have a nervous system).
(II) *Restricting the prevalence of epigenetic inheritance of the effects of stress*: We expect that mechanisms restricting the transgenerational transmission of the effects of stress to the next generation have evolved in animals during the Cambrian, so it is likely that intergenerational inheritance of stress responses will be more common in animals with non-associative learning or limited associative learning than in animal manifesting UAL. Although the effects of neuro-hormonal destabilization following extreme trauma or persistent stress still have epigenetic, transgenerational effects (e.g., Jawaid et al. 2018) such inheritance is likely to have been more common during the early Cambrian. We therefore predict that widespread epigenetic inheritance will be found in sessile animals with rudimentary brains and in short-lived animals with central nervous systems such as Planaria and nematodes.
(III) *Co-evolution of active forgetting and UAL*: We expect to find evidence for the co-evolution of the GRNs underlying active forgetting mechanisms with those underlying UAL, and trace back the origins of these GRNs to the co-evolutionary dynamics of the Cambrian era.

### Summary of the predictions

The claim that UAL is a natural cluster, in the sense that the different elements group together in UAL animals (claim 1), leads to the predictions that:

(a) The five elements of UAL (see Part I) are ontogenetically correlated. Developmental studies are expected to show that the development of one element facilitates or enables the development of one or more of the other elements.
(b) The five elements of UAL are phylogenetically correlated. Finding that a species has evolved one of the elements of UAL raises the probability that the species has evolved the whole UAL package.
(c) The five elements of UAL are medically correlated. Brain injuries that affect one element will not leave the other elements completely unaffected, but may leave more limited forms of learning unaffected.

The claim that UAL is a transition marker for the transition to experiencing systems (claim 2) leads to the predictions that:

(d) Experimental protocols such as backward masking that selectively switch off conscious perception in humans, leaving unconscious perception in place, will selectively switch off UAL, while leaving more limited forms of learning in place.
(e) Blindsight patients will be unable to perform UAL on stimuli presented in the blind region of the visual field, but they may be capable of more limited forms of learning.
(f) The neural signatures of subjective experience in humans, whatever they turn out to be, will be correlated with UAL.

The claim that UAL (and thus conscious experience) is possessed by most vertebrates, some arthropods, and the coleoid cephalopods (claim 3) leads to the predictions that:

(g) Animals from these phyla will be able to discriminate between novel compound stimuli, exhibit the capacity for trace conditioning, second-order conditioning, reversal learning, spatial learning and pattern completion, evaluate action options that are context-dependent and make adaptive motivational trade-offs.
(h) In conditions equivalent to masking in humans the animals will fail in these tasks, although they will be still able to show simpler forms of learning.

The claim that the evolution of UAL had trade-offs and led to the evolution of new coping mechanisms (the most easily testable part of claim 4), leads us to predict that:

(i) Molecular changes in GRNs underlying the stress response and those underlying AL and UAL will be correlated.
(j) Epigenetic inheritance in animals exhibiting UAL will be more constrained than epigenetic inheritance in animals with simpler forms of learning.
(k) Molecular changes in the GRNs underlying AL and UAL and those underlying active forgetting will be correlated.

## Discussion

One of the main attractions of the UAL approach is that it tries to cut through the noise of disagreement to find a hidden consensus. The idea is that, for all the disagreement, there is a consensus that a list of hallmarks of consciousness are jointly sufficient (given the actual laws of nature) for consciousness. Much of the disagreement is about which, if any, of those jointly sufficient conditions are also necessary. However, there are some approaches to studying consciousness that are clearly opposed to the UAL framework, and we will close with a brief discussion of these.

First, the UAL approach, while compatible with many specific theories about the nature of consciousness, is not compatible with higher-order thought (HOT) theories of the type defended by Carruthers (2000), Rosenthal (2005) and LeDoux (2019). The core commitment of a HOT theory is that consciousness involves a system representing its own mental states. Such theories posit that a mental state becomes phenomenally conscious when it becomes the object of a higher-order representation. A higher-order theorist will simply deny that the hallmarks of consciousness listed in Part I suffice for consciousness. They will maintain that a crucial extra ingredient—an ability to form representations of one’s own mental states—is needed. This is not the place for a detailed discussion of HOT theories. It is enough to say that they cannot provide convincing evidence that this extra ingredient is in fact needed, and that they face significant problems explaining why this extra ingredient should be needed (Block 2011; Carruthers 2017).

For a higher-order theorist, the UAL approach casts the net too widely, counting as conscious many animals that (in their view) are probably not conscious. But some may have the opposite concern. Could the UAL approach be locating the transition to experiencing systems much too late in evolutionary history?

Some radical views regard conscious experience as a basic property of all life, or even all matter. The former view is “biopsychism” (a term coined by Haeckel 1892, p. 483; for a recent exposition see Reber 2018), the latter “panpsychism” (Goff 2019). Such views are logically compatible with the UAL framework, since their defenders can agree that the list of hallmarks identified in Part I are sufficient for consciousness. They will just think that the hallmarks go far beyond what is necessary. We’re not sure what a pan/biopsychist would say about our proposed transition marker, and we don’t want to prejudge that question. If they disagree with our proposed transition marker and want to suggest a more basic one—one so minimal it can be possessed by unicellular organisms—they are welcome to do so. We can then evaluate the evidence that this alternative transition marker does indeed require a set of capacities that suffice for consciousness.

We should stress again a point made in Part I: according to the UAL framework, UAL is a positive marker of consciousness. It allows us to make a positive case for consciousness in some animals, but it is not intended to tell us which animals are not conscious. An approach based on positive markers leaves open the possibility that some animals have a form of consciousness that fails to generate the positive marker. So, readers who want from the UAL framework a definitive answer to the question “which animals are not conscious?” will be disappointed.

However, a similar issue arises for all major evolutionary transitions and innovations (unless it is assumed that the capacity in question appeared all at once, in a saltational manner). When an evolutionary transition occurs, grey areas that elude definition are very likely to be found. The assignment of a mode of being (living, subjective experiencing, reflecting) to the entities in the grey areas will always be controversial. It is not clear, for example, whether we can call a very simple autopoietic system (e.g., a chemoton that does not have a self-replicating polymer) alive. However, there are clear-cut cases on either side of the boundary. A bacterium is alive, whereas a single molecule of sugar is not alive—because life is a systemlevel property, and none of the hallmarks of life are displayed by individual molecules in isolation.

We think the same is true for consciousness. There can be a consensus about systems that have sufficient conditions for consciousness, and a consensus about entities that do not have any member of any empirically supported set of sufficient conditions, despite substantial disagreement about borderline cases. The boundaries between modes of being are vague. Yet the study of systems which inhabit the grey areas is of crucial importance for the understanding of the evolutionary history of both life and consciousness.

## Figures and Tables

**Table 1:** We provide references, mostly books and reviews, very sparingly here because of the broad scope of each of the topics; more references for the behavioural signatures column are given in part II of the paper

	Neurophysiological and cognitive mechanisms	Behavioural signatures	Phenomenological manifestations
Binding/unification	Integration of information through synchronous and sequential binding mechanisms (Baars 2005a, b; Dehaene 2014)	Discrimination between complex patterns (Couvillon and Bitterman 1988)	Different features of an object are bound together into a single percept (e.g. we experience a blue box, not blueness and boxness) (Searle 2004)
Global accessibility	Multimodal integration of inputs from sensory, evaluative and memory systems (Dehaene 2014)	Memory and evaluation of, as well as discrimination among, multimodal patterns in multimodal discrimination learning (Mansur et al. 2018; Telles et al. 2017)	Experience is a single, integrated whole (e.g. we experience sights, smells, sounds, emotions and memories together) (Searle 2004)
Flexible value system	Integrative systems for valuing and revaluing different stimuli and for weighing different needs against each other (Morsella 2005)	Reversal learning; second order conditioning; decision making in situations of conflict (Hadar and Menzel 2010; Gewirtz and Davis 2000)	Feelings of pleasure and displeasure; felt emotions; moods (Metzinger 2003; Searle 2004)
Selective attention and exclusion	Attentional networks (Petersen and Posner 2012)	Habit formation and autopilot behaviour; damages to learning under distracting conditions (Dehaene 2014)	Some elements of experience are the focus of attention; others fade into the background (Searle 2004)
Intentionality	Hierarchical mapping of body and world (Feinberg and Mallatt 2016)	Goal-directed behaviour based on goal representation (Dickinson 2008)	Experience represents the world and the subject’s own body. Metzinger 2003)
Integration over time	Working memory; fragile short-term memory (Baddeley 1986)	Capacity for trace conditioning; delayed match-to-sample; ability to learn from video sequences (Lucas et al. 1981; Bangasser et al. 2006)	The ‘specious present’; the ‘stream’ of consciousness (James 1890)
Embodiment and agency	Mechanisms of top-down cognitive control over motor output (Zhang et al 2016)	Exploration guided by motor-sensory-motor (MSM) loops (Ahissar and Assa 2016)	The sense of embodied, goal-directing agency (James 1890; Merleau-Ponty 1945; Merker 2007)
Self-other registration	Interaction of neural models of self, body and motivated action, generating egocentric representations of the moving animal in space (von Holst and Mittelstadt 1950; Merker 2007)	Damage to self-model (e.g. following stroke) leads to feelings of disowning one’s body parts (Vallar and Ronchi 2009)	The feeling of ownership of one’s experiences; the structure of experience as a “point of view” on the world (Metzinger 2003; Merker 2007)

## Footnotes

1 Gánti’s chemoton model, which consists of three coupled subsystems—an autocatalytic metabolic engine, a self-assembling membrane subsystem, and a digital polymer system—forms a functional unit manifesting all the properties of life.

2 For more on the concept of a “mode of being”, see Ginsburg and Jablonka (2020). Ginsburg and Jablonka also identify a third major mode of being, the “rational-reflective” mode. This three-way distinction between living, conscious and rational-reflective modes of being is intended to map approximately on to Aristotle’s distinction between nutritive, sensitive and rational souls.

3 We say “minimal” because we take it that some elements of some presentations of the global workspace, such as broadcast to mechanisms of verbal report and planning (e.g. Dehaene and Changeux 2011, p. 209) are not necessary for consciousness.

4 In Ginsburg and Jablonka (2019), agency, embodiment and registration of self are listed under one heading, because of their substantial overlap.

5 This line is often attributed to Einstein, but there is in fact no clear provenance for the remark.

6 The presentation of UAL in Ginsburg and Jablonka (2019) listed only components 1–3, but they also discussed flexible, easily rewritable associations with value and the ability to bridge temporal gaps, as essential attributes of UAL (see, for example, pp. 234, 235, 368, 402).

7 Evidence for second-order conditioning in rats, rabbits and pigeons is reviewed by Gewirtz and Davies (2000). Second-order conditioning was found in goldfish by Farr and Savage (1978). Evidence for compound conditioning in rats, rabbits, pigeons and goldfish is briefly reviewed in Couvillon and Bitterman (1988), who go on to present evidence of compound conditioning in honey bees. For studies of trace conditioning see Lucas et al. (1981) (pigeons), Bangasser et al. (2006) (rats), Moyer et al. (2015) (rabbits); Rodríguez-Expósito et al. (2017) (goldfish).
